# Supplementary material for: STIL binding to Polo-box 3 of PLK4 regulates centriole duplication
Source: eLife. 2015 Jul 18;4:e07888. doi: 10.7554/eLife.07888 (PMC4530586; doi:10.7554/eLife.07888)
Supplement: Supplementary file 1. — Molecular mass determination by lateral diffusion measurements. DOI: http://dx.doi.org/10.7554/eLife.07888.020 [file elife07888s001.docx]

**Supplementary File 1: Molecular mass determination by lateral diffusion measurements.**

| Protein molecule | Theoretical molecular mass [kDa] ^a^ | Diffusion coefficient *D* [m^2^s^-1^]^b^ | Experimental molecular mass  [kDa]^c^ |
| --- | --- | --- | --- |
| PLK4-PB3 | 9.6 | 1.13∙10^-10^ | 9.6 |
| PLK4-PB3/STIL-CC | 13.4 | 9.64∙10^-11^ | 15.3 |
| GB1^d^ | 6.2 | 1.22∙10^-10^ | 7.6 |

^a^ Calculated from the amino acid sequence for a protein monomer

^b^ Determined by BPP-LED NMR experiments

^c^ Calculated from the diffusion coefficient assuming spherical, rigid molecules

^d^ Control protein: Immunoglobulin binding domain of streptococcal protein G (Gronenborn et al., 1991)
